# Can Physical Activity in Immersive Virtual Reality Be Attractive and Have Sufficient Intensity to Meet Health Recommendations for Obese Children? A Pilot Study

**DOI:** 10.3390/ijerph17218051

**Published:** 2020-11-01

**Authors:** Jacek Polechoński, Katarzyna Nierwińska, Barbara Kalita, Piotr Wodarski

**Affiliations:** 1 Institute of Sport Sciences, The Jerzy Kukuczka Academy of Physical Education in Katowice, 40-065 Katowice, Poland; 2 Department of Physiological and Medical Sciences, The Jerzy Kukuczka Academy of Physical Education in Katowice, 40-065 Katowice, Poland; k.nierwinska@awf.katowice.pl; 3 Metabolic Diseases Clinic, the Voivodeship Specialist Clinic Complex in Katowice, 40-038 Katowice, Poland; barbara.kalita@gmail.com; 4 Department of Biomechatronics, Faculty of Biomedical Engineering, Silesian University of Technology, 41-800 Zabrze, Poland; piotr.wodarski@polsl.pl

**Keywords:** immersive virtual reality, virtual reality, health-oriented physical activity, intensity of physical activity, active video games, omnidirectional treadmill

## Abstract

Immersive virtual reality (IVR) is a technology that blurs the line between the physical world and a digital environment. Using appropriate pointing devices, it is possible to engage in physical activity (PA). The main aim of the study was to assess the attractiveness and intensity of physical exercise while playing active video games (AVGs) in IVR on an omnidirectional treadmill by obese children and to present the results compared to health recommendations (PA). It was also assessed whether the AVGs storyline can effectively motivate the participants to undertake locomotor activity by increasing the intensity of their effort (moving in a limited space vs. having to follow a set route). Eleven children aged 8 to 12 years with diagnosed obesity participated in the experiment. The attractiveness of PA was assessed with a questionnaire, while the intensity of exercise was estimated on the basis of heart rate. The answers show that AVGs are attractive and more enjoyable for the respondents than conventional video games. All participants declared their willingness to practice this form of PA. The intensity of PA of obese children during two games was high but during the game where the player was supposed to follow a set route, it was significantly higher (83.3 ± 9.2% HRmax) than during the game whose storyline assumed moving in a limited space (77.4 ± 9.8% HRmax). Due to the high intensity of PA while playing the AVGs studied, it can be assumed that obese children can benefit for their health if the games are used on a regular basis. However, further research is needed to verify this thesis.

## 1. Introduction

Today, childhood obesity has reached epidemic levels in both developed and developing countries. It is generally accepted that the main cause of overweight and obesity in children and adolescents is excessive calorie intake. The decreased levels of PA also plays an essential role in this health concern [1]. One of the causes of the sedentary lifestyles of contemporary children and youth is excessive time spent on screen media [2,3]. Video games are especially attractive to children. Unfortunately, numerous studies have demonstrated that overweight and obesity may be attributable to a long time spent playing video games [4,5,6,7,8,9]. An advantageous alternative to conventional computer games is offered by active video games (AVGs), sometimes called exergames. Recent years have seen a growing interest in this form of entertainment as a source of PA [10]. Research has been conducted to assess the intensity of PA of various AVGs in the context of health recommendations for PA. Research results vary depending on the type of game and console. Some authors indicated that the intensity of PA is rather low [11], while others found that its levels are moderate or high [12,13,14,15]. There are reports that AVGs can be useful in combating overweight and obesity as they help increase daily dose PA and that playing these games can reduce BMI in children [16,17]. These studies concern PA in a non-immersive VR, where the user controls the computer application displayed on the screen using body movements.

The last few years have seen the development of immersive virtual reality (IVR), the technology that blurs the line between the physical and the digital world by creating a sense of being present in the virtual environment for the user. The goal of IVR is to completely immerse the user inside the computer-generated world, giving the impression to the user that he/she has stepped inside the synthetic world. This can be achieved by using the technology of Head-Mounted Display (HMD). Immersive VR with HMD uses HMD to project VR just in front of the eyes and allows users to focus on the display without distraction. A virtual environment is a place where a person can perform various movements and practice various forms of PA. Therefore, IVR is gaining more and more interest among physiologists, coaches, and physiotherapists [18,19].

There are reports that IVR is likely to reduce the intensity of various types of pain by distraction from thinking about this problem [20,21,22,23]. All of the VR interventions for pain management are solely based on cognitive distraction. Perhaps a similar effect could be achieved for alleviating the discomfort that accompanies intense PA, which may be particularly effective in individuals with reduced physical fitness and physical capacity, such as people with obesity, who could be encouraged by IVR to exercise longer. This thesis is partly supported by a study by Baños et al. [24] who demonstrated that VR is useful for distracting overweight and obese children during exercise. Furthermore, Matsangidou et al. [25] demonstrated that virtual reality reduces the negative sensations related to performing isometric exercises, thus increasing the likelihood of performing them for a longer time. Furthermore, this environment is attractive and can motivate AVGs users to engage in physical exercise [26,27].

The prerequisite for the health benefits of PA in IVR is a sufficient level of intensity, which according to the WHO recommendations [28], should be moderate or high. It is difficult to meet this prerequisite without the use of appropriate pointing devices. The use of conventional controllers and HMDs is usually limited to the movement of the upper limbs. Greater opportunities to engage in PA are offered by the combination of IVR with exercise machines. The following devices are beginning to appear on the market: stationary bikes, treadmills, and even rowing, flying, and diving simulators, which are coupled with the equipment supporting IVR applications. With these devices, the player can perform more energetic movements as he or she is protected against an uncontrolled fall. The devices also encourage more intensive training as they help involve larger muscle groups. Research on the evaluation of PA using IVR exercise machines also began with the arrival of this type of technology on the market. The first attempts are being made to analyze locomotor activity on IVR treadmills [29,30,31,32]. Studies are beginning to examine the exercise load in people using different exercise machines and show that the intensity of physical exercise while playing AVGs in IVR is so high that a beneficial effect on health can be expected [33] and that the PA in IVR can be even more intense than during conventional training sessions [34].

Among the commercially available exercise machines that can be used with IVR, particularly interesting devices are omnidirectional treadmills, which allow not only for locomotion but also for versatile body movements. According to research on gait analysis, this type of equipment can be used in training for overweight people and those with obesity, because locomotive motion performed on the treadmill is more sliding-like compared to the natural gait, and thus they transfer less load to the lower limb joints [29].

Bearing in mind the above-mentioned arguments and that most of the research related to human motor activity in IVR concerns adults [35], we decided to conduct PA-related research on children with obesity using an omnidirectional treadmill. The main aim of the study was to evaluate the intensity of PA and the rating of perceived exertion (RPE) in children with obesity playing two different AVGs in IVR and to present the results obtained as compared to health recommendations for PA. It was also assessed whether the AVGs storyline can effectively motivate the participants to undertake locomotor activity by increasing the intensity of their effort (moving in a limited space vs. having to follow a set route). An attempt was also made to find whether in the opinion of the examined children, AVGs in IVR are attractive and can offer an alternative to conventional video games and other forms of PA.

## 2. Materials and Methods

Eleven children aged 8 to 12 years with obesity were included in the study: seven boys (age 10.1 ± 1.7 years, body weight 67.4 ± 9.5 kg, body height 148.1 ± 8.8 cm, BMI 30.7 ± 2.9 kg/m^2^) and four girls (age 8.8 ± 1.0 years, body weight 49.8 ± 9.1 kg, body height 137.8 ± 10.7 cm, BMI 26.2 ± 3.1 kg/m^2^). They were all patients of the Metabolic Diseases Clinic, the Voivodeship Specialist Clinic Complex in Katowice. Only the children with the pediatrician’s and parents’ consent were allowed to participate in the experiment. The participants also had to meet the following inclusion criteria: good general health status, no medical contraindications to participate in the study (in particular no previous epileptic episodes and motion sickness), no physical limitations (e.g., injuries) that could prevent PA in IVR, and no medication that could affect the heart rate. Furthermore, none of the participants had previously experienced immersive virtual reality based on the device used in the research. However, children had a lot of experience with conventional video games. All children declared that they played video games several times a week for a few hours.

**Figure 1:**
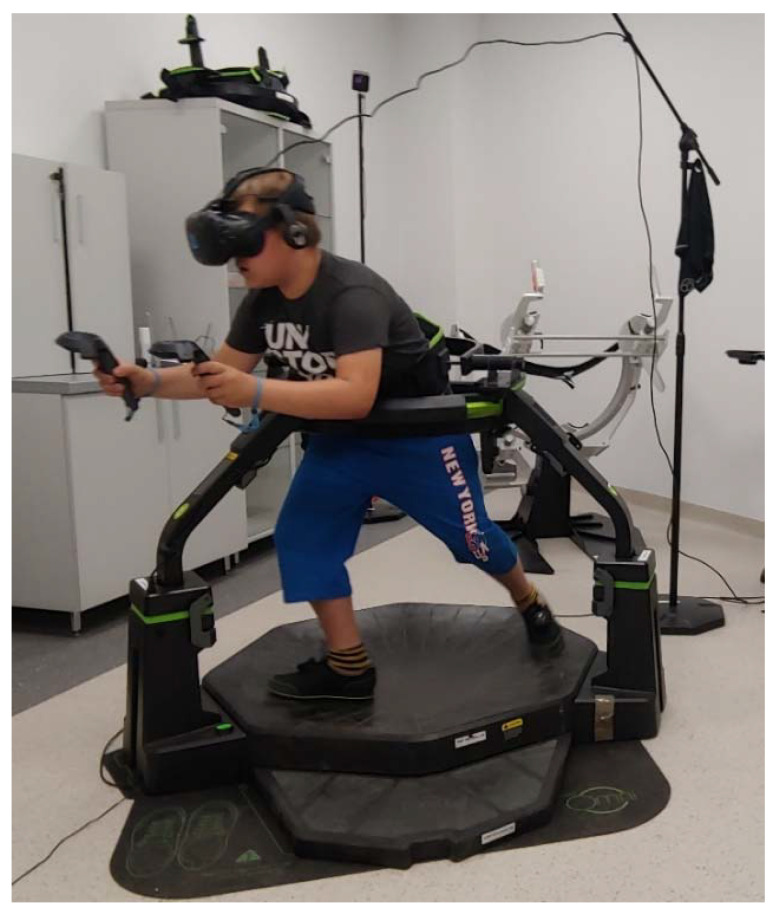
Test stand: participant on an OMNI omnidirectional treadmill playing an active video game (AVG) in immersive virtual reality (IVR).

The experiment included two 15-min AVGs sessions in IVR (Core Defense and Travar Training OPS) available on the Omniverse digital distribution platform (https://omniverse.virtuix.com) interspaced with a 30-min break. The games were played on an OMNI omnidirectional treadmill (Virtuix Inc., Austin, TX, USA) (Figure 1). Before each game, the researcher adjusted the system to the participant’s body height, explained the purpose of the game, and explained how to operate and move in VR. Next, there was a 2-min trial game, followed by the actual game, which lasted 15 min. The players could terminate their participation in the research at any time. Children first played Core Defense and then Travar Training OPS. They started with Level 1.

**Figure 2:**
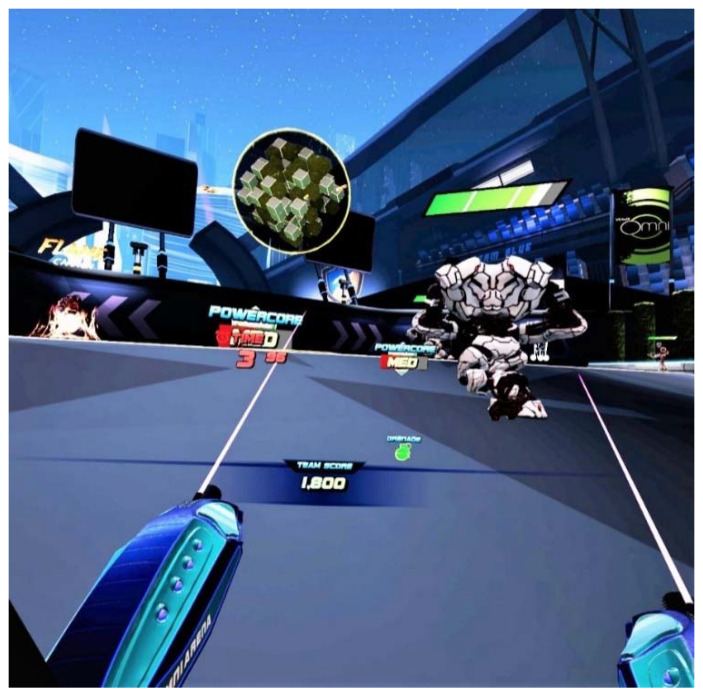
Gameplay of Core Defense game: print screen.

**Figure 3:**
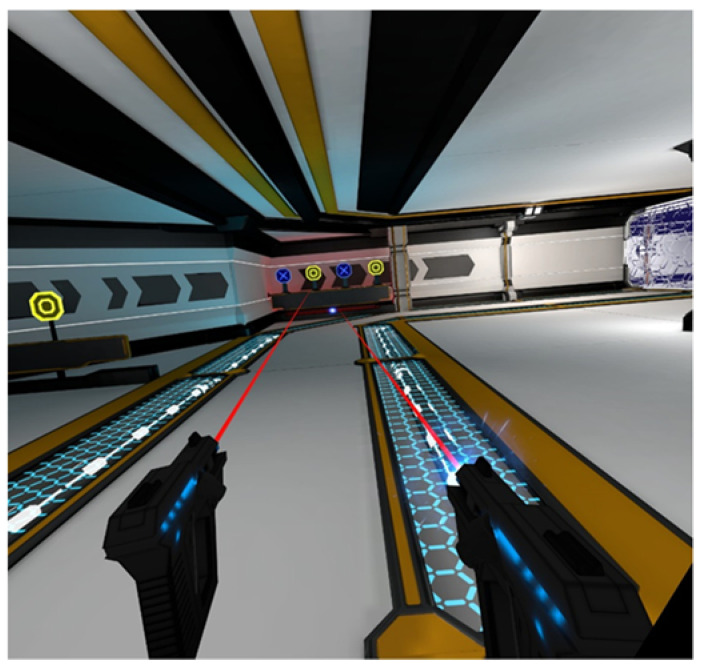
Gameplay of Travar Training OPS game: print screen.

Both games used in the research involved performing locomotive motion in IVR. In the first game (Core Defense), the player moves in a limited space (arena) and decides whether and at what moment to move. The aim of the game is to eliminate moving or stationary targets (robots) by shooting them with a laser weapon held in both hands (Figure 2). In the second game, Travar Training OPS, the player needs to cover the set route, in a way that forces the player to perform the locomotive motion. The game consists of destroying moving and stationary targets with a laser weapon held in both hands (Figure 3). Projection of VR images and upper limb movements as the player moved on the treadmill were controlled by HMD and HTC VIVE controllers (HTC Corporation, New Taipei, Taiwan) compatible with the OMNI platform.

The subjective assessment of the attractiveness and fun of playing AVGs in IVR using the exercise machine tested and their competitiveness compared to conventional video games and other forms of PA was verified using a questionnaire prepared by the authors containing 8 questions with yes/no scale (Table 1). The children were also asked which game seemed more attractive for them.

While playing AVGs, the heart rate (HR) was monitored using the Vantage V heart rate monitor (Polar Electro Oy, Kempele, Finland). The intensity of physical exercise was determined based on the mean maximum heart rate percentage (% HRmax) obtained by each participant during the experiment. Prior to the experiment, the HRmax value of the participant was calculated from the formula developed by Tanaka et al. [36] (208 − 0.7 × age). According to Mahon et al. [37], the equation can closely predict HRmax in children. Exercise load was estimated based on the classification of the intensity of physical exercise proposed by the American College of Sport Medicine [38]. According to the classification, average HR (HRavg) < 64% HRmax means low intensity, 64% HRmax ≤ HRavg < 77% HRmax—moderate intensity, and HRavg ≥ 77% HRmax—high intensity. The data obtained using this method were compared to the criteria of health recommendations for the intensity of aerobic exercise, according to which the exercise of at least moderate intensity is considered beneficial for health (≥64% HRmax) [28]. The total time of HR recorded during a 15-min exercise on the exercise machine was also evaluated using six intensity zones: <50% HRmax, 50–59% HRmax, 60–69% HRmax, 70–79% HRmax, 80–89% HRmax, and ≥90% HRmax.

**Figure 4:**
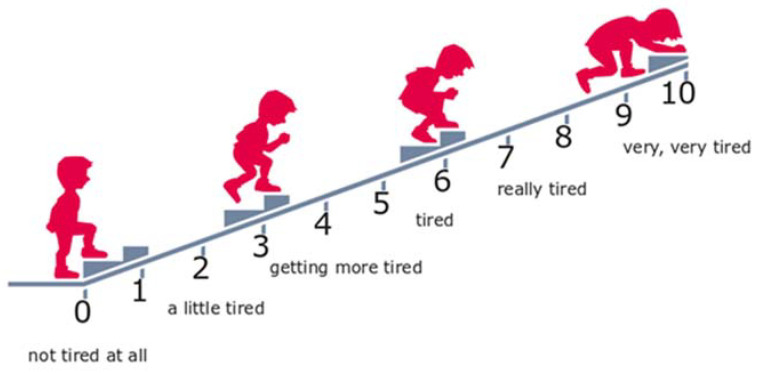
Children’s OMNI Scale of perceived exertion used in the experiment.

The perceived exertion was estimated using the 10-degree Omni scale for children [39]. After completion of the game, the participant marked, on a pictorial scale, the level of fatigue they experienced during PA in IVR (Figure 4). RPE was compared with the results of the objective assessment of the intensity of physical effort.

The study procedures were reviewed and approved by the Research Ethics Committee of the Jerzy Kukuczka Academy of Physical Education in Katowice (protocol number 9/2018). All participants took part in the study voluntarily and could discontinue their participation at any time. Informed written consent was obtained from parents or legal guardians of children.

Statistical calculations were performed using Statistica v.13 (TIBCO Software Inc., Palo Alto, CA, USA). The analysis of measurement data was conducted using basic descriptive statistics. The results of the survey are presented in percentages. Arithmetic means, standard deviations, and differences of mean results were calculated. The data were tested for normal distribution using the Shapiro–Wilk test, whereas the significance of differences was evaluated using the Wilcoxon test. Spearman’s rho correlation analysis was used to assess relationships.

## 3. Results

### 3.1. Attractiveness of AVGs in IVR Using an OMNI Treadmill and Their Competitiveness Compared to Conventional Computer Games and Other Forms of PA According to the Participants

The survey showed that AVGs in IVR were attractive for all the examined children. This is also confirmed by the answers to the second question. It turns out that after the first contact with AVGs on the OMNI treadmill, children would like to explore other similar games. However, the answer to the third question shows that for almost all children (91%) AVGs are more enjoyable than conventional video games because they are more fun to play. The vast majority of respondents (73%) would replace conventional video games for AVGs in IVR. All children agreed that AVGs in IVR on the OMNI treadmill are more realistic than conventional computer games. Each research participant declared that if they had the appropriate hardware and software they would engage in PA in IVR and would recommend this form of entertainment to others. For most respondents (64%), AVGs in IVR on OMNI treadmill were even more attractive than conventional forms of PA such as movement games and playing, team sports, running, cycling, swimming, etc. (Table 1). To the question “Which of the two games was more attractive to you?” all children indicated Travar Training OPS.

### 3.2. Intensity Level of PA in the Context of Health Recommendations and Perceived Exertion When Playing AVGs in IVR

**Figure 5:**
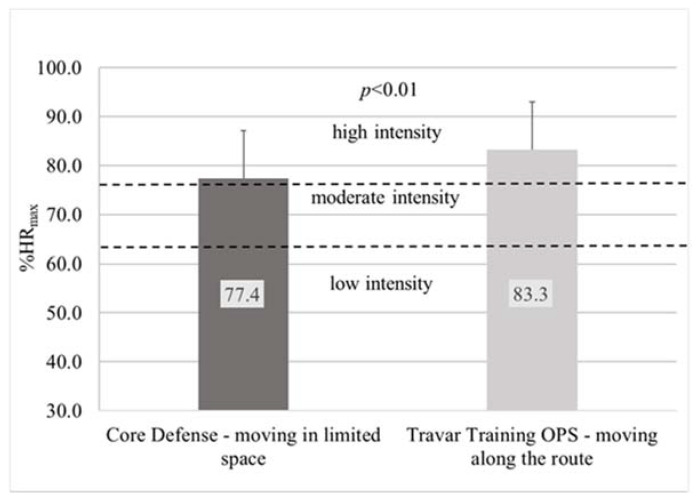
Average intensity of PA of obese children during PA in IVR on an omnidirectional treadmill depending on the type of video game.

Measurements using the heart rate monitor showed that the mean heart rate of the examined children playing Core Defense on the OMNI omnidirectional treadmill was 155.5 ± 19.1 bpm and was significantly lower (*p* < 0.01) than that observed during Travar Training OPS game (167.0 ± 18.0 bpm). A similar statistically significant relationship was found by analysis of the average percentage of maximum heart rate (% HRmax). The estimated parameter for people playing Core Defense was 77.4 ± 9.8% HRmax and was significantly lower (*p* < 0.01) than for playing Travar Training OPS (83.3 ± 9.2% HRmax) (Figure 5). Both games were therefore characterized by high intensity of physical exercise recommended for health benefits.

**Figure 6:**
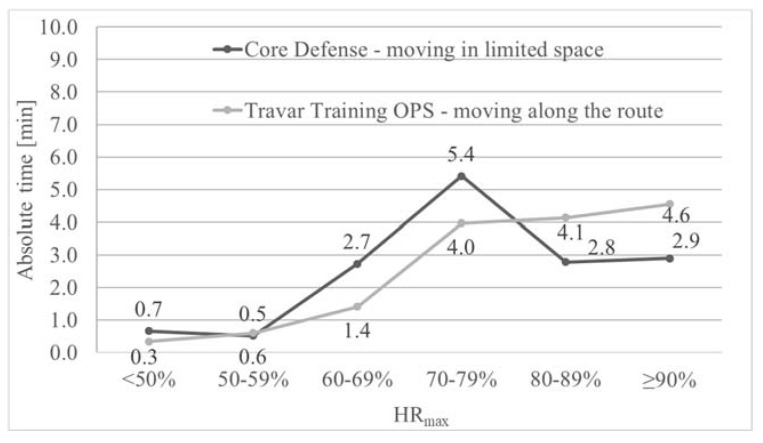
Average total time spent in different heart rate zones by obese children during PA in IVR on an omnidirectional treadmill depending on the type of video game.

The heart rate monitor readings revealed that during a 15-min playing Core Defense, the heart rate of the participants was equal or higher than 60% Hrmax for an average of 13.8 min (92.1% of the test duration) While playing Travar Training OPS, the time was even longer and was 14.1 min (93.8% of the test duration). Analysis of the time spent during both games in individual heart rate zones revealed the greatest discrepancy in the 60–69% zone (1.3 min) and the 70–79% zone (1.4 min). However, these differences were not statistically significant (Figure 6).

**Figure 7:**
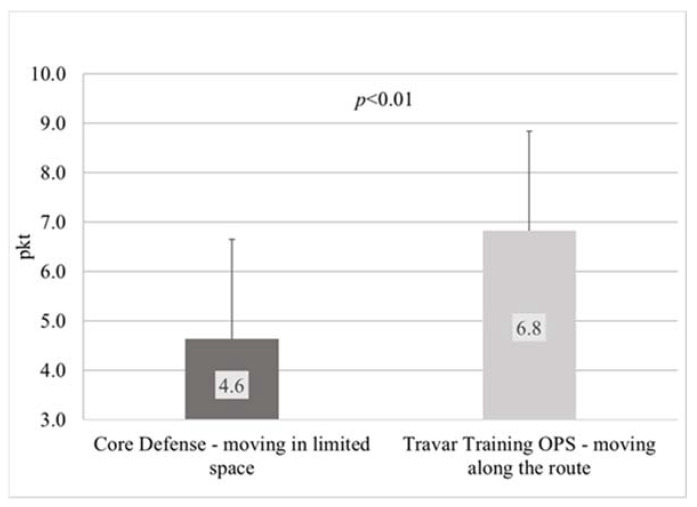
Rating of perceived exertion (10-degree scale) in obese children during PA in IVR on an omnidirectional treadmill depending on the type of video game.

In the subjective assessment of the intensity of physical exercise made by the participants on a 10-degree scale, playing Core Defense was also significantly less intense (*p* < 0.01) than Travar Training OPS. The perceived exertion while playing the former game was rated by children at 4.6 ± 2.0 points, and during the latter, it was rated at 6.8 ± 2.8 points (Figure 7). However, no significant correlation was found between HR and RPE (Core Defense: ρ = 0.3, Travar Training OPS: ρ = 0.3).

## 4. Discussion

Undoubtedly, the AVGs in IVR played on an omnidirectional treadmill were attractive to obese children participating in our study, as evidenced by individual answers to the survey questions. As AVGs in IVR are more enjoyable than conventional video games for most respondents, it is likely that soon they will become competitive with classic computer games played in a seated position, thus offering a healthy alternative for children. This is all the more likely because the AVGs used in our study were rather low-budget products that cannot compete in terms of computer graphics with popular games from major computer or console developers. Therefore, their high assessment by the respondents is a good predictor. It can be assumed that the improved graphic design of AVGs can increase the positive experience of users. This assumption is consistent with a study by Farič et al., [40] who showed that the quality of computer graphics has a particularly positive effect on the fun of playing IVR. The positive perception of playing AVGs in IVR was also demonstrated by other authors. Dębska et al. [33] analyzed the level of satisfaction and intensity of PA of healthy adults playing on a similar treadmill and using the Icaros flight simulator on a 1–7 Likert scale. The authors found that the users rated the fun of PA in IVR higher while playing on a treadmill (5.74 ± 0.86 points) compared to playing a flight simulation (5.60 ± 0.86 points) although in the former case the intensity of physical exercise was high and in the latter case it was low. A similar relationship was observed in our study. Travar Training OPS was more attractive for all participating children and was characterized by a higher level of intensity of PA. This proves that high physical exercise during AVGs in IVR does not reduce their attractiveness. Furthermore, Farrow et al. [41] showed that IVR can significantly increase the pleasure of exercising on a cycle ergometer during high-intensity interval training. This is consistent with previous studies which confirmed that stationary cycling in a virtual environment gives users more satisfaction than in conventional conditions [34,42]. This is probably related to the gamification effect [43]. The high attractiveness of PA in IVR using exercise machines may encourage players to undertake regular exercises. Many studies have demonstrated that satisfaction is an important predictor of participation in PA, regardless of the age and health status of the participants [44,45,46,47,48,49]. Due to the great pleasure that physical exercise in a virtual environment offers to users, AVGs can potentially become a viable alternative for conventional forms of PA. For most of the obese children we examined, AVGs played on the OMNI treadmill were more attractive than conventional forms of PA such as movement games and playing team sports, running, cycling, swimming, etc. However, this can be also considered a threat. From the point of view of participation in social life and integration with peers, total replacement of the above-mentioned forms of physical exercise for that practiced in IVR would be inadvisable for young people. Therefore, AVGs should be rather complementary to conventional forms of PA.

In our study, the intensity of PA of obese children playing both games was high (HRavg > 77% HRmax). Similar results were reported by Dębska et al. [33], who examined adults exercising on an omnidirectional treadmill. Therefore, it must be assumed that in the case of both AVGs, the intensity of physical exercise was sufficient to provide health benefits if this form of PA is practiced on a regular basis [28]. As shown in our research on the group of obese children, during the game characterized by the necessity to cover a designated route, which forced the player to make a locomotive motion (Travar Training OPS), the intensity of PA was significantly higher than during a game in which the player moved in a limited space (Core Defense). This is indicated by objective measurements recorded with a heart rate monitor. This is a valuable guide that can be used by manufacturers of AVGs, who can modify the intensity of their games and influence player engagement by designing specific game scenarios depending on the needs. Also based on the subjective perception of the participating children, playing Travar Training OPS is more intense than Core Defense, which proves that children can distinguish between the intensity of PA during AVGs based on their own experience of PA in IVR. However, the results of the correlation analyses indicate that HR-RPE correlations were not reliable for estimation during AVGs.

The ability of obese children to perform high-intensity PA in IVR for a long time is probably related to the effect of diverting their attention from bodily sensations that represent a discomfort during conventional exercises. The potential of VR to increase distraction from bodily sensations in overweight children and those with normal weight during PA was evaluated by Baños et al. [24]. Their research concerned walking on a mechanical treadmill in natural settings and in a virtual 3D graphical environment. The participants focused more on internal information during the conventional walking test than when walking in VR. The authors also showed that the distraction effect was stronger in overweight children than in those with normal weight. VR increased pleasure during exercises and therefore the respondents preferred physical exercise in a virtual environment. According to the authors, virtual reality is useful for distraction and can help overweight and obese children to enjoy the exercises. Similar effects may be caused by e.g., sound stimuli. The research by Deforche and De Bourdeaudhuij [50] showed that distracting overweight children by music can have a positive effect on extending the running distance and exercise intensity. In the case of IVR, diverting the attention of the player from bodily sensations is undoubtedly more effective as more senses are stimulated. The research results quoted suggest that during training in a virtual environment, obese children are likely to perform intense exercise, forgetting about the discomfort that may accompany such exercise. Furthermore, increased physical exercise performance in IVR was linked by Yao and Kim [51] with high psychological arousal of players and their level of presence during the game. The authors examined young adults playing a stationary cycling video game on a cycle ergometer based on two levels of immersion (flat screen vs. HMD). The travel distance within the game was recorded and analyzed. The results showed that subjects who played IVR using HMD covered a longer travel distance and also felt a higher level of presence and psychological arousal. According to the authors, this provides evidence that video games based on IVR can increase user’s physical exercising performance. This assumption is also consistent with a study by McClure and Schofield [34] conducted in a group of college students, showing that the participants declared the willingness to continue the IVR exercises on a cycle ergometer for a longer period of time compared to similar conventional training.

In the context of the health impact of the AVGs used in our studies, attention should also be paid to the form and structure of movements performed by users. During PA in IVR, the children performed mainly movements of upper limbs (aiming at the target) and lower limbs (locomotion). Undoubtedly, the high intensity of the games was mainly due to the locomotive motion performed on the OMNI omnidirectional treadmill. Movement on such a treadmill is slightly different from a natural gait. A detailed comparative analysis of both forms of gait was presented by Jochymczyk-Woźniak et al. [29] who demonstrated that due to the noticeable sliding component of the locomotive motion and the longer support phase during the treadmill gait cycle, such walking can reduce the load on the lower limb joints compared to normal gait. This is particularly important for people who are overweight and obese, as excessive load on the lower limbs contributes to the occurrence of osteoarthritis [52]. The safety of moving on the treadmill is also ensured by its design. One of the components of the device is a belt that stabilizes the user and protects against falling (Figure 1). In the context of PA for obese people, preventing from uncontrolled loss of balance seems critical. An increased risk of falls is observed in obese middle-aged and older adults [53,54] but there is an established relationship between the prevalence of obesity in children and the frequency of limb injuries [55].

In conclusion of the problems discussed in the present study, related to the attractiveness of AVGs and the potential offered by IVR to encourage obese children to engage in appropriately intensive and thus healthy PA, it seems that entertainment in a virtual environment can be used in the process of primary and secondary obesity prevention. However, further research is needed to verify this thesis.

Due to the pilot character of this study, several limitations should be indicated. A relatively small group of children was examined as a result of the quite strict inclusion criteria, as described in the Participants chapter. A more accurate method of estimating the intensity of physical exercise should be considered to be used in the future, e.g., a method based on indirect calorimetry. In our study, we used % HRmax because the aim of the study was also to obtain information from children about the attractiveness of PA in IVR. There was a concern that the masks used in calorimetry may cause discomfort and lead to the understated subjective assessment of the participant. However, it may be problematic for children to simultaneously wear HMD and an ergospirometry mask. We are also aware of the fact that the present research can only be used to conclude about the potential health benefits of PA in IVR on the OMNI treadmill for obese children. In order to verify this, it would be necessary to assess the effects of long-term training, which should encourage further research. Another limitation is that the typical validated PA enjoyment scale was not used, but in our pilot studies we wanted to obtain information on the attractiveness of AVGs in IVR compared to typical video games and other PAs. Therefore, a new questionnaire was created.

## 5. Conclusions

As our pilot studies show, the intensity of PA in obese children playing AVGs in IVR on an omnidirectional treadmill is high and depends on the plot of the game. This is confirmed by both objective measurements and rating of perceived exertion. The game where the player needs to follow a designated route is more stimulating to performing locomotive motion and therefore more intense PA compared to the player moving in a limited space (arena). This is a valuable indication for manufacturers of AVGs promoting PA in IVR. It seems that with a well-designed VR and a properly selected game scenario, it is possible to influence the player’s engagement and modify the intensity of physical exercise based on the locomotive motion. In the opinion of the children surveyed, AVGs in IVR are attractive and appear to be a beneficial alternative to conventional video games played in an unhealthy sitting position. Due to the high intensity of PA while playing the AVGs tested, it can be assumed that obese children can benefit for their health if the games are played on a regular basis. Perhaps in the future, AVGs in IVR will become a method to reduce body fat in obese children. However, further research is needed to verify this thesis.

## Figures and Tables

**Table 1:** Attractiveness of active video games (AVGs) in immersive virtual reality (IVR) using OMNI treadmills and their competitiveness compared to conventional computer games and other forms of physical activity (PA) according to the obese children.

Question	Yes/Agree	No/Disagree
1. Were the games in IVR using the OMNI treadmill attractive to you?	100%	0%
2. Would you like to explore other games available on the OMNI platform?	100%	0%
3. Do the AVGs in IVR on the OMNI treadmill give you more fun than conventional computer games?	91%	9%
4. Would you exchange conventional video games for AVGs in IVR?	73%	27%
5. Are the AVGs in IVR on the OMNI treadmill more realistic than conventional computer games?	100%	0%
6. Would you engage in PA in IVR if you had the right hardware and software?	100%	0%
7. Would you recommend practicing PA in IVR to others (friends, family)?	100%	0%
8. Are the AVGs in IVR on the OMNI treadmill more attractive to you than conventional forms of PA (movement games and playing, team sport, running, cycling, swimming, etc.)?	64%	36%
